# Immunogenicity of a Fap2 peptide mimotope of *Fusobacterium nucleatum* and its potential use in the diagnosis of colorectal cancer

**DOI:** 10.1186/s13027-018-0184-7

**Published:** 2018-04-02

**Authors:** Leonardo A. Guevarra, Andrea Claudine F. Afable, Patricia Joyce O. Belza, Karen Joy S. Dy, Scott Justin Q. Lee, Teresa T. Sy-Ortin, Pia Marie S. P. Albano

**Affiliations:** 1 Department of Biochemistry, Faculty of Pharmacy, University of Santo Tomas, Manila, Philippines; 2 Research Center for Natural and Applied Sciences, University of Santo Tomas, Manila, Philippines; 3 Benavidez Cancer Institute, University of Santo Tomas Hospital, Manila, Philippines; 4 Department of Biological Sciences, College of Science, University of Santo Tomas, Manila, Philippines

**Keywords:** Fap2 protein, *Fusobacterium nucleatum*, ELISA, Colorectal cancer, Immunodiagnostics

## Abstract

**Background:**

The role of *Fusobacterium nucleatum* Fap2 protein in the development of colorectal cancer has recently been explained. Fap2, when bound to the human inhibitory receptor, TIGIT, inhibits the cytotoxic activity of natural killer (NK) cells against cancer cells, thus, allowing proliferation of the latter eventually leading to tumor growth. The aim of the study was to identify the immunogenicity of a peptide mimotope of the Fap2 protein and to determine the reactivity of colorectal cancer patients’ sera against the mimotope.

**Methods:**

Immunogenic epitope of the Fap2 protein of *F. nucleatum* was selected using the B-cell epitope prediction of the Immune Epitope Database and Analysis Resource (IEDB). The immunogenicity of the synthetic peptide mimotope of the Fap2 protein was determined in animal models and reactivity of colorectal cancer patients’ sera against the mimotope was done by indirect ELISA.

**Results:**

Results show that the selected peptide mimotope, with sequence TELAYKHYFGT, of the outer membrane protein Fap2 of *F. nucleatum* is immunogenic. Increase in the absorbance readings of peptide-immunized rabbit sera was observed starting Week 1 which was sustained up to Week 10 in the indirect ELISA performed. Colorectal cancer cases (*n* = 37) were all reactive in an ELISA-based analysis using the mimotope as the capture antigen.

**Conclusions:**

In this study, we identified an immunogenic epitope of the Fap2 protein of the *Fusobacterium nucleatum*. We demonstrated the reactivity of serum of histopathologically confirmed CRC patients in a peptide-capture indirect ELISA which may serve as proof of concept for the development of CRC diagnostics.

## Background

The composition of the gut microbiome has recently been implicated in the development of colorectal cancer (CRC) [1]. Dysbiosis, a condition characterized by a pathological imbalance in the microbial community, is shown to contribute in the growth and progression of colorectal tumors [2]. Several studies reported the high association of microorganisms belonging to the proteobacteria, such as *Pseudomonas, Helicobacter,* and *Acinetobacter*, with colon cancer [3]*.* The most current is that of *Fusobacterium nucleatum*, whose role in CRC progression has recently been described [4].

*F. nucleatum* is a non-spore forming, non-motile, gram-negative, spindle-shaped opportunistic anaerobic bacteria that can be found in the oral cavity and the gastrointestinal tract [5]. Its cell envelope is composed of outer and inner membranes flanking a periplasmic space and outer membrane proteins which comprise a third of its mass [6]. Originally identified as an oral commensal and a periodontal pathogen, *F. nucleatum* has recently been associated with several human illnesses which include adverse pregnancy outcomes, cardiovascular disease, rheumatoid arthritis, respiratory tract infections, Lemierre’s syndrome, Alzheimer’s disease, and gastrointestinal disorders including CRC [7]. The association of *F. nucleatum* with CRC development is attributed to the ability of patients’ infected cancer cells to inhibit the ability of the immune system to attack tumoral cells [7, 8].

Fap2 protein is a 390-kilodalton protein encoded by the *Fap2* gene of *F. nucleatum* [9]. It is an outer membrane protein composed of 3692 amino acid which is identified to induce apoptosis in human lymphocytes [9, 10]. Recently, Fap2 protein has been shown to be involved in the binding of *F. nucleatum* to cancer cells and to interact with the immunoglobulin and ITIM domain (TIGIT) receptor mainly expressed on NK, Treg, CD8+ and CD4+ T cells [11]. The binding of Fap2 to TIGIT was found to inhibit the activity of natural killer (NK) cells against the tumor cells, thus causing the growth and progression of CRC [12].

Given this role of the *F. nucleatum* Fap2 protein in CRC tumorigenesis and the lack of a reported Fap2 immunogenic epitope in the Immune Epitope Database (IEDB) Resource Analysis (www.iedb.org), this study, therefore, sought to search for an immunogenic epitope of the *F. nucleatum* Fap2 protein to test the immunogenicity of a peptide mimotope in vivo and to measure the reactivity of plasma samples from confirmed CRC patients and clinically healthy controls to the mimotope.

## Methods

### Immune epitope prediction and peptide mimotope synthesis

Prediction of the immune epitope of *Fusobacterium nucleatum* Fap2 protein was done in silico using the Immune Epitope Database (IEDB) Analysis Resource. The primary sequence of the Fap2 protein was taken from the National Center for Biotechnology Information (NCBI) Protein Database. The amino acid sequence of the epitope was selected using B-cell epitope prediction platform of the IEDB. Candidate immunogenic epitope was selected based on its antigenicity, surface accessibility, and hydrophilicity using Kolaskar & Tongaonkar Antigenicity Scale and Emini Surface Accessibility Scale. The predicted immunogenic mimotope was synthesized by Genscript (New York, USA).

### Immunization

Immunogen was prepared by conjugating the peptide mimotope to bovine serum albumin (BSA) following the methods of Coligan et al. [13]. Peptide conjugation in BSA (10:1) was done in the presence of glutaraldehyde in borate buffer (pH 10) [13]. The BSA-conjugated peptide solution was reconstituted with PBS to produce a 1 mg/mL stock solution, which was then dialyzed against 4 l of water for 24 h.

Immunogenicity of the peptides was tested in 10- to 12-week-old white male New Zealand rabbits. Animal care and immunization were done following the protocols described by Bio-Synthesis (http://www.biosyn.com/) and as approved by the Institutional Animal Care and Utilization Committee (IACUC) of the University of Santo Tomas, Philippines. Six rabbits were randomly assigned to negative control (*n* = 2) and experimental (*n* = 4) groups. Each rabbit from the experimental group was immunized with 1.0 mL solution containing 100 μg of peptide distributed to five subcutaneous sites and two intramuscular sites. The immunogen was prepared by reconstituting the previously prepared peptide-BSA stock solution with PBS emulsified with Complete Freund’s Adjuvant (Sigma). Four booster doses of the same amount of peptide emulsified with Incomplete Freund’s Adjuvant (Sigma) were given at two weeks interval. Rabbits in the negative controls were given the same treatment except that the solutions used in the immunization did not contain the peptide mimotope.

### Immunogenicity assay

Immunogenicity of the synthetic peptide mimotope of the Fap2 predicted epitope was analyzed by detecting presence of anti-peptide mimotope antibody in the sera of rabbits. Blood was collected through the marginal ear vein before immunization to serve as baseline, and after immunization at weekly intervals for 10 weeks for the immunogenicity assay. Serum was separated from the cells by centrifugation of the collected blood at 3400 × g. Collected serum was transferred to Eppendorf tubes and stored at − 20 °C until use.

Presence of anti-peptide mimotope antibody was detected by indirect ELISA. Wells were coated with 50 μL of coating buffer (0.05 M carbonate buffer, pH 9.6) containing 10 μg of peptides and blocked with 100 μL of blocking solution (2% gelatin in 0.01 M PBS containing 0.05% Tween 20). Fifty microliters of 1:100 diluted rabbit sera were then added to each well after washing with wash buffer (0.01 M PBS containing 0.05% Tween 20) and incubated for 1 h at 37 °C. The wells were washed again to remove the unbound rabbit antibodies. HRP-conjugated goat anti-rabbit antibody (Genscript, New York) was used to detect presence of anti-peptide mimotope rabbit antibody using TMB as substrate.

### Reactivity assay

Reactivity of sera collected from confirmed CRC cases (*n* = 37) and clinically healthy case-matched controls was also determined by indirect ELISA as previously mentioned. Patients’ sera were also diluted 1:100 and the anti-peptide mimotope human antibody was detected using HRP-conjugated anti-human antibody (Koma Biotech, Seoul, Korea). Protocols on the use of left-over plasma samples were approved by the Ethics Review Board of the University of Santo Tomas Hospital.

### Statistical treatment and analysis

Comparison of the mean absorbance readings from the indirect ELISA of negative control and experimental groups from baseline up to 10 weeks after the initial immunization was done by multivariate analysis using IBM SPSS Statistics version 20. *P*-values less than 0.05 were considered statistically significant.

The ELISA cut-off values were computed based on the formula described by Frey et al. [14]. Values higher than the computed cut-off were assessed as reactive while those that were lower were evaluated as non-reactive.

## Results

In this study, selection of a candidate immune epitope was done using in silico analysis tools embedded in the Immune Epitope Database and Analysis Resource (IEDB). The sequence of the predicted immunogenic epitope was TELAYKHYFGT which is located at the 3596th to 3606th position of the Fap2 protein. Antigenicity and surface accessibility scores were 1.028 and 1.198, respectively, which were above the cut-off values 0.998 and 1.000, respectively. The synthetic peptide mimotope of the selected immunogenic epitope was also reported to be soluble in ultrapure water, 0.1 M PBS pH 7.1, and DMSO, at a gross peptide concentration of less than 15 mg/mL.

**Fig. 1:**
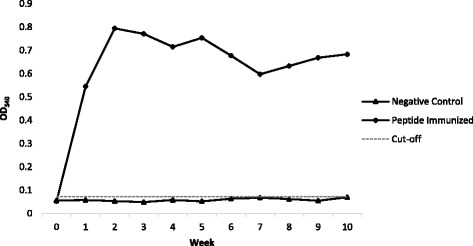
Immunogenicity of a Fap2 Peptide Mimotope. Indirect ELISA absorbance readings of peptide immunized group as compared to the negative control and computed cut-off value

Immunogenicity of the synthetic peptide mimotope was evaluated by monitoring the weekly anti-peptide antibody titer in the animal models immunized with the peptide. Figure 1 presents the weekly mean absorbance readings of the negative control and peptide immunized groups.

The mean absorbance of the baseline blood samples of the peptide immunized and negative control groups were 0.052 and 0.055, respectively. These readings were below the computed cut-off value of 0.072. An increase in the absorbance was observed in the peptide-immunized group but not in the negative control group starting a week after the first immunization. A constant increase in anti-Fap2 titer was observed from Week 1 to Week 10 of immunization of the experimental rabbits. Independent *t*-test showed no significant difference in the absorbance readings of baseline blood samples collected from the normal control and peptide immunization group (*p*-value = 0.387). However, multivariate analysis revealed statistically significant increase in the absorbance readings starting Week 1 after immunization as compared to the negative control (*p*-values< 0.02).

**Fig. 2:**
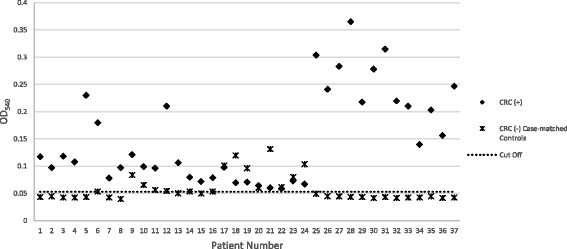
OD_540_ Readings of Colorectal Cancer Patients and Case-matched Healthy Controls. OD_540_ values higher than the computed cut-off value of 0.05284 were assessed as reactive. All CRC patients were reactive while 25 of the 37 case-matched controls have values lower than the cutoff

After confirming the immunogenicity of the synthetic peptide, we tested for the reactivity of plasma samples from histologically confirmed colorectal cancer cases and their age- and sex- matched clinically healthy controls to the peptide by indirect ELISA. Figure 2 presents the OD_540_ readings of CRC positive sera and their case-matched controls in contrast to the cut-off value of 0.05284.

All cases (*n* = 37; 100%) showed reactivity to the Fap2 peptide mimotope. Twelve of the controls (32%) also tested positive for IgG against the peptide (Table 1). These results gave a sensitivity of 100% and a specificity of 68%. The positive predictive value and the negative predictive value are 76% and 100%, respectively.

**Table 1:** Seroreactivity to *Fusobacterium nucleatum* Fap2 Peptide Mimotope of Colorectal Cancer Cases and Clinically Healthy Controls

Seroreactivity to Fap2 Peptide Mimotope	CRC Cases		Healthy Controls		Total	
	*n*	%	*n*	%	*n*	%
Reactive	37	100%	12	32	49	66%
Non-reactive	0	0	25	68	25	34%
Total	37	100%	37	100%	74	100%

## Discussion

In silico analysis tools provide a valuable platform in predicting and selecting candidate peptide sequences that can be used for immunodiagnostics and immunotherapeutics [15]. They have improved and greatly accelerated epitope design, which is a crucial step in vaccine as well as in diagnostic development [16]. When existing databases provide limited information on desired immunogenic epitopes, such as the case of the Fap2 protein of *F. nucleatum*, several immune epitope prediction tools, which are based on the physico-chemical properties of a portion of a polypeptide chain, can be used [17]. These physico-chemical properties include antigenicity, surface accessibility, and hydrophilicity based on the frequency of existing amino acids in the primary sequence of a target antigen [17–19].

In this study, we have shown in vivo the immunogenicity of the in silico-predicted immunogenic epitope of Fap2 antigen. The statistically significant increase in the absorbance readings in the ELISA of the mimotope-immunized rabbits, a pattern which was not observed in negative control group, is indicative of the production of an anti-peptide IgG.

Peptides have the ability to induce an immune response and prompt the cell to produce antibodies [20]. Synthetic peptides can excellently mimic proteins because they are exact copies of protein fragments which are responsible for the protein’s activity [21]. Synthetic peptide immunogens can induce an immune response by mimicking the activity of the subunit of the whole protein antigen and be used as a promising tool in vaccine and immunodiagnostic tool development [22]. Because of the simplicity, ease of use, and target specificity of utilizing peptide mimotopes, this procedure is preferred over other existing methods [22, 23]. Although one major challenge in using peptides as immunogen is its weak immunogenic property, this can be solved by conjugating it with carrier proteins such as BSA and keyhole limpet hemocyanin [24].

Synthetic peptides mimicking immunogenic epitopes of protein antigens can initiate and activate the immune response by binding to either the Class I or Class II major histocompatibility complex (MHC) [25]. In 2005, Wang et al. demonstrated the ability of peptide mimotopes prepared from MHC Class I library of baculovirus to bind to T-cell and secret interleukin-2 [26]. In the same year, Gevorkian et al. were able to elicit polyclonal antibodies against a cell surface antigen of *Mycobacterium tuberculosis* in rabbits using a 15-mer chemically synthesized peptide [27]. Buchwald et al (2005), in their search for a potential vaccine against pneumococcus, observed that a 12-mer peptide elicited production of antibodies in mice models which were then seroprotected during the infection challenge [28]. The anti-tumor activity of peptide mimotopes through the production of anti-HBJ127, a tumor suppressing anti-CD98 heavy chain antibody, was observed by Saito et al. [29].

Synthetic peptides are also used in ELISA in the diagnosis of diseases [30]. Antibodies present in serum of immunized or previously infected individuals reacts to the peptides in immunoassays [31–33]. This was also observed in our experiments when we used the peptide as the capture antigen in the ELISA performed to detect presence of anti-peptide antibody in the sera of peptide-immunized rabbits as well as patients with colorectal carcinoma – a disease associated with the presence of harmful microbiota, such as *F. nucleatum*, in the gut.

The human gut is home to a complex system of microorganisms which play a vital role in the host’s health homeostasis [2]. Recent studies suggest the role of the gut microbiota in the development in colorectal cancer due to their ability to interfere in the host’s inflammatory and immunomodulatory activities, which subsequently favor carcinogenesis and tumorigenesis [33–36]. Among the recently studied and implicated in colorectal carcinoma development is *F. nucleatum* [37, 38]. This bacterium has also been associated with chemoresistance of cancer cells during treatment [38]. Hence, there is the need to develop methods for their detection as well as determine the anti-*F. nucleatum* titer that is associated with CRC.

*F. nucleatum*’s outer membrane protein Fap2 is identified to adhere to host’s cell surface and induce pro-inflammatory and pro-carcinogenic response [39]. Its association to the development of colorectal cancer and its role in immune evasion was first reported by Kaplan et al. [40] when they observed that *F. nucleatum* proteins induce lymphocyte apoptosis. This was further supported by Mima et al. [8] when they observed that the number of *F. nucleatum* cells in CRC cases was observed to be inversely associated with the density of CD3^+^ T-cells. Recently, proposed roles and molecular mechanisms of *F. nucleatum* outer membrane proteins in immune evasion have been reported. The ability of *F. nucleatum* to bind colorectal adenocarcinoma cells was reported previously [41]. It was also observed that the binding of Fap2 to human inhibitory receptor TIGIT protects the cancer cells from the immunosurveillance activity of NK cells, which, in effect, would allow the cancer cells to grow and proliferate [11]. These studies serve as impetus in understanding the role and mechanism of Fap2 protein in the colorectal carcinogenesis.

The ability to identify the immunogenic epitopes of the Fap2 of *F. nucleatum* is relevant in developing immunodiagnostic and therapeutic methods for CRC. The immunogenicity of and the reactivity of the CRC patients’ plasma samples to the peptide mimotope can serve as starting point in developing methods in the screening and detection CRC.

## Conclusions

We have identified an immunogenic epitope of the Fap2 protein of *F. nucleatum*. The peptide TELAYKHYFGT located at the 3596th to 3606th amino acid of the outer membrane protein Fap2 of the CRC-associated pathogen *F. nucleatum* proved to be immunogenic in animal models. Seroreactivity of CRC patients against the peptide mimotope in an indirect ELISA was also observed.

Identifying immunogenic epitopes of CRC-associated pathogens such as *F. nucleatum* may serve as impetus in the development of vaccine and immunodiagnostic tools against colorectal cancer. Future studies may use results from this study to pursue a pathogen-targeted control of cancer similar to the human papilloma virus (HPV) or as a biomarker for detection and determination of risks of cancer development similar to Eppstein Barr virus.

## Abbreviations

BSA: bovine serum albumin
CRC: Colorectal cancer
ELISA: Enzyme-linked immunosorbent assay
HRP: horseradish peroxidase
IACUC: Institutional Animal Care and Utilization Committee
IEDB: Immune epitope database
IgG: Immunoglobulin G
NCBI: National Center for Biotechnology Information
NK: Natural killer
PBS: phosphate buffered saline
TIGIT: T cell immunoglobulin and ITIM domain (TIGIT)
TMB: 3,3′,5,5′-Tetramethylbenzidine
